# Editorial: Wheat: from nutrition to cultivation and technology

**DOI:** 10.3389/fnut.2025.1563397

**Published:** 2025-02-07

**Authors:** Bojana Voučko, Elena Bartkiene, Marianna Rakszegi, João Miguel F. Rocha

**Affiliations:** ^1^Laboratory for Cereal Chemistry and Technology, Department of Food Technology, Faculty of Food Technology and Biotechnology, University of Zagreb, Zagreb, Croatia; ^2^Institute of Animal Rearing Technologies, Faculty of Animal Sciences, Lithuanian University of Health Sciences, Kaunas, Lithuania; ^3^Department of Food Safety and Quality, Veterinary Academy, Lithuanian University of Health Sciences, Kaunas, Lithuania; ^4^Cereal Breeding Department, Agricultural Institute, Centre for Agricultural Research, Martonvasar, Hungary; ^5^Universidade Católica Portuguesa, CBQF – Centro de Biotecnologia e Química Fina – Laboratório Associado, Escola Superior de Biotecnologia, Porto, Portugal

**Keywords:** wheat, cereals, bread, breadmaking, grain

Wheat accounts for one-fifth of the total food calories and protein consumed by the global population, ensuring food security worldwide (1, 2). Climate change poses significant challenges to global wheat production, necessitating adaptation strategies to ensure food security (3). Increased awareness of the environmental changes and health benefits of plant-based diets have directed current research of wheat in human nutrition to sustaining abundant wheat production while enhancing its technological and nutritional quality. Addressing these challenges requires multidisciplinary approaches, integrating advanced technologies, and investments in research and innovation (4).

A long-term experiment on breadmaking wheat cultivars in different Mediterranean environments of Maçãs et al. determined their resilience to climate change. The authors presented breeding programs which are focused on both yield and technological quality, adapted to dry and hot environments, which are expected to become more common due to climate change. Results showed certain cultivars, such as Antequera, showed better resilience to different environmental conditions, performing well in terms of both yield and quality traits across a range of climates. The interaction between cultivar and environment showed significant effects on wheat yield and quality traits, indicating the importance of testing cultivars across different environments to assess their resilience to climate change.

Further on, Johansson et al. showed the contribution of introgression of alien genes into wheat to improve food security in the face of climate change. This paper provided a comprehensive review of how alien introgression into wheat contributes to yield, resistance to disease and pests, functional and nutritional quality, and the development of novel wheat-based food products, using both traditional and emerging plant breeding methods.

The review by Gupta et al. highlighted the nutritional importance of wheat through a comprehensive review of biofortification of staple crops as a promising strategy to address the micronutrient deficiencies, which pose a public health challenge globally. A meta-analysis of per capita daily intake indicated that despite being a staple food, wheat falls short of meeting recommended daily allowances for iron (Fe) and zinc (Zn). The authors provided an overview of the concept, trends, approaches, bioavailability, health impact, and policy framework to address widespread micronutrient deficiencies through wheat biofortification, a sustainable and cost-effective strategy.

The paper by Kucharska et al. provided an insight into the importance of wheat and wholegrain cereal consumption in the adult population. The paper offers an in-depth analysis of dietary fiber intake, examining the sources of fiber and the relationship between dietary choices and fiber intake by age and sex. The main source of fiber in the adult population diet were cereal products 44.1% followed by vegetables. Only 7.5% of fiber was obtained from whole grain bread, acknowledging once more that consumption of wholegrain cereals could have a significant effect on improvement of public dietary habits.

The paper from Stemler et al. aimed to analyze the lipid composition and lipase-induced reactions in cake batter and baked cakes, to establish a connection between specific lipase reactions and their macroscopic effects on cake quality. The main finding of this paper is that the substrate specificity of different lipases, is the key factor in determining their effectiveness in improving cake batter and baking quality. The research offers new insight into development of the clean-label enzyme use in bakery products other than bread.

This Research Topic from *Frontiers in Nutrition*, section *Nutrition and Food Science Technology*, highlights the importance of breeding programs on developing resilient wheat varieties, expanding genetic diversity and adaptation to climate change, wheat biofortification to address micronutrient deficiencies, importance of cereals in diverse high-fiber diets to mitigate diet-related non-communicable diseases, as well as use of clean label improvers in production. Besides contributing to improved nutritional and functional qualities in wheat, these advancements are crucial for food security under changing climate conditions. Wheat represents a fundamental food crop that has played a crucial role in human civilization for millennia (5). Ensuring global food safety in the future means applying a multidisciplinary approach to wheat cultivation and technology, in accordance with the changing nutrition, consumer preference and climate change.
